# Impact of renin-angiotensin system inhibitors continuation versus discontinuation on outcome after major surgery: protocol of a multicenter randomized, controlled trial (STOP-or-NOT trial)

**DOI:** 10.1186/s13063-019-3247-1

**Published:** 2019-03-05

**Authors:** Matthieu Legrand, Emmanuel Futier, Marc Leone, Benjamin Deniau, Alexandre Mebazaa, Benoît Plaud, Pierre Coriat, Patrick Rossignol, Eric Vicaut, Etienne Gayat, for the STOP-OR-NOT study investigators

**Affiliations:** 1 AP-HP, GH St-Louis-Lariboisière, Department of Anesthesiology and Critical Care and Burn Unit, St-Louis Hospital, Assistance Publique-Hopitaux de Paris, Paris, France; 2 University Paris Diderot, Paris, France; 3 UMR INSERM 942, Institut National de la Santé et de la Recherche Médicale (INSERM), Lariboisière Hospital, Paris, France; 4 F-CRIN INI-CRCT network, Nancy, France; 5 Département de Médecine Périopératoire, Anesthésie Réanimation Hôpital Estaing, CHU Clermont-Ferrand & Université Clermont Auvergne, CNRS, Inserm, Clermont-Ferrand, France; 6 Service d’Anesthésie et de Réanimation, Hôpital Nord, Aix Marseille Université, APHM, Marseille, France; 7 Département d’Anesthésie-Réanimation, La Pité-Salpétrière, Université Paris Descartes, Paris, France; 8 Inserm, Centre d’Investigations Cliniques-Plurithématique 14-33, Inserm U1116, CHRU Nancy, Université de Lorraine, Nancy, France; 9 Unité de recherche Clinique, GH St-Louis-Lariboisère-Fernand Widal, Université Paris Diderot, Paris, France

**Keywords:** ACE inhibitors, ARB, Strategy, Outcome, Complications, Acute kidney injury, Surgery, Mortality

## Abstract

**Background:**

Chronic treatment of hypertension or heart failure very often includes an angiotensin-converting enzyme inhibitors (ACE-Is) or angiotensin receptor blockers (ARBs) as renin-angiotensin system inhibitors (RASi) treatments. To stop or not to stop these medications before major surgery remains an unresolved issue. The lack of evidence leads to conflicting guidelines with respect to RASi management before major surgery. The purpose of this study is to evaluate the impact of a strategy of RASi continuation or discontinuation on perioperative complications in patients undergoing major non-cardiac surgery.

**Methods:**

This is a multicenter, open-labeled randomized controlled trial in > 30 French centers. In the experimental group, RASi will be continued while the treatment will be stopped 48 h before the surgery in the control arm. The primary endpoint is a composite endpoint of major complications after surgery. An endpoint adjudication committee will review clinical data and adjudicate efficacy endpoints while blinded to the assigned study drug group. Main analysis will be by intention-to-treat comparing the composite outcome measure at 28 days in the two groups. A total of 2222 patients are planned to detect an absolute complications difference of 5%.

**Discussion:**

The results of the trial should provide robust evidence to anesthesiologists and surgeons regarding management of RASi before major non-cardiac surgery.

**Trial registration:**

ClinicalTrials.gov, NCT03374449. Registered on 11 December 2017.

**Electronic supplementary material:**

The online version of this article (10.1186/s13063-019-3247-1) contains supplementary material, which is available to authorized users.

## Background

More than 200 million major surgical procedures are performed annually worldwide. Many of these patients have co-morbidities including hypertension and/or heart failure [1, 2]. Chronic treatment of hypertension and/or heart failure very often includes a renin-angiotensin system inhibitor (RASi, angiotensin-converting enzyme inhibitors [ACE-Is] or angiotensin receptor blockers [ARBs]. To stop or not to stop these medications before major surgery remain an unresolved issue [3–7]. It is most likely that the strategy regarding management of RASi in the perioperative setting has an important impact on perioperative complications [8–10].

The lack of evidence leads to conflicting guidelines with respect to RASi management before major surgery. While French guidelines are to stop RASi patients with hypertension to avoid profound anesthetic-drugs-induced hypotension, international guidelines differ. The American Heart Association task force states that continuation of RASi perioperatively is reasonable (class IIa recommendation, level of evidence: B). They also recommend that if RASi therapy is withheld, it should be restarted as soon as feasible (level: C) [11]. These recommendations are based on few data suggesting an increased risk of overall complication after RASi discontinuation [12, 13] in the perioperative setting, while intraoperative hypotension can be managed with available vasoactive drugs during anesthesia [14]. The RAS acts on the vascular function and promotes thrombosis [15–17]. Modulating the RAS is therefore likely to impact global outcome after major surgery. The purpose of this study is to evaluate the impact of a strategy of RASi (i.e. ACE-Is or angiotensin receptor antagonists) continuation or discontinuation on perioperative complications in patients undergoing major non-cardiac surgery.

## Methods

### Design

**Fig. 1:**
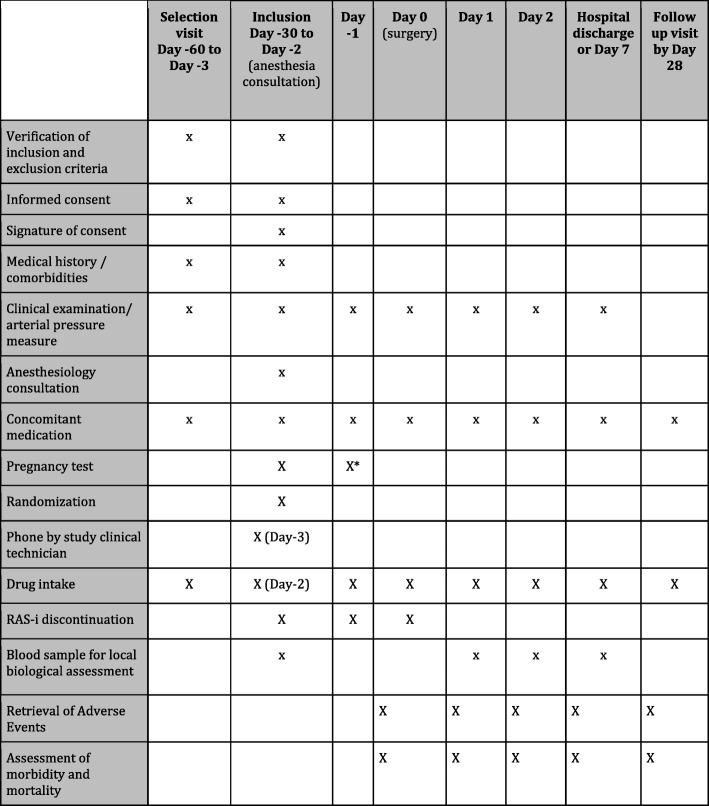
SPIRIT schematic schedule of enrolment, interventions, and assessments

The protocol was designed in accordance to the guidelines [18] (Fig. 1). This is a multicenter, open-labeled randomized controlled trial (RCT) in > 30 French centers. The randomization is performed at the CRU “Lariboisière-St Louis” and stratified by center and on the chronic heart failure status (New York Heart Association [NYHA] stage ≥ 2). The randomization list has been developed by a different biostatistician from the biostatistician who will conduct the final analysis within the CRU “Lariboisière- St Louis.” The list is inserted into the web-based software and then forwarded to the sponsor’s quality insurance team for validation. The patients are randomized on the day of written consent obtainment by web (CleanWeb) software, which assigns the patient a randomization number. Randomization is performed by blocks of four or eight with allocation concealment.

**Fig. 2:**
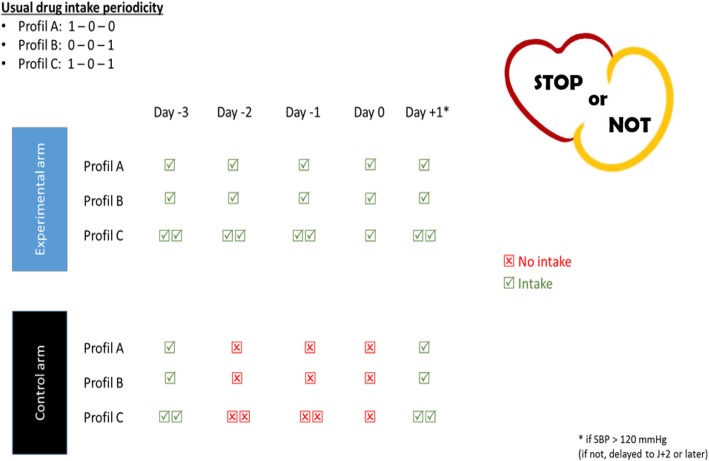
*Scheme* of drug intake according to the treatment arm (experimental arm with continuation of the treatment or control arm with withholding of the drug 28 h before surgery). Profiles A, B, and C refer to the number of drug intakes across a day

The randomization will be performed after the anesthesiology consultation and informed written consent has been obtained (Additional file 1). After randomization, a prescription indicating the treatment regimen in accordance to the arm in which the patient has been assigned will be edited automatically, printed, handed out, and explained to the patient. A phone call will be performed three days before surgery by a clinical study technician to ensure good understanding of the medication protocol (exact schedule of stopping of the chronic medication) and proper application to the protocol. In the experimental group, RASi will be continued, including on the day of the surgery, while the treatment will be stopped 48 h before surgery in the control arm (Fig. 2). Stopping the treatment 48 h before the surgery was proposed to avoid any remnant effect on the RAS blockage after stopping the treatments [19]. All patients will receive a leaflet in which they will record the discontinuation or continuation of the RASi. In this leaflet, the patients of the group: continuation of the RASi will report the adhesion to the protocol (i.e. treatment intake). The information collected in the leaflet will be add to the patients’ medical records and then collected by the clinical research assistant. We suggest the treatment will be resumed as soon as possible based on postoperative hemodynamic and renal status and availability of enteral route after surgery. The date of re-institution is indeed being collected. Patients having combined general anesthesia together with neuraxial anesthesia or peripheral nerve block could be enrolled.

#### Inclusion criteria

Inclusion criteria were as follows: patients requiring major surgery defined as a surgery with an expected duration of > 2 h from the surgical incision and a postoperative hospital stay of least three days [20, 21]; age ≥ 18 years; signed informed consent; chronically treated (> 3 months before surgery) with RASi; and women of childbearing potential must agree to use adequate contraception.

#### Exclusion criteria

Exclusion criteria were as follows: emergency surgery (surgical treatment needed within 48 h); hyperkalemia (serum potassium level > 5.5 mmol/L) at the time of the anesthesiology consultation; patients for which death is deemed imminent and inevitable or patients with an underlying disease process with a life expectancy of < 1 month; patients with severe chronic renal insufficiency as defined by estimated glomerular filtration rate < 15 mL/min/1.73 m^2^ or requiring renal replacement therapy; patient with preoperative shock (defined by the need for vasoactive drugs before surgery); and lack of social insurance.

### Main objective and primary endpoint

The main objective is to evaluate the impact of RASi continuation or withholding on postoperative complications in patients undergoing major non-cardiac surgery.

The primary endpoint is a composite endpoint of all-cause mortality and major postoperative complications within 28 days after surgery, defined as one or more of the following:

- death;
- postoperative cardiovascular events (acute myocardial infarction, arterial or venous thrombosis, stroke, acute pulmonary edema, postoperative cardiogenic shock, acute severe hypertension crisis, severe cardiac arrhythmia requiring therapeutic intervention [22]);
- postoperative episodes of sepsis;
- postoperative respiratory complication (defined by the need for re-intubation and/or non-invasive ventilation for respiratory failure);
- unplanned intensive care unit admission or readmission;
- acute kidney injury (based on the serum creatinine item of the KDIGO criteria, baseline serum creatinine is preoperative value) and/or hyperkalemia (serum potassium level > 5.5. mmol/L requiring intravenous therapeutic intervention); and
- surgical complication (need for reoperation for any reason and radiologic interventions for abscess drainage).

### Secondary objectives and secondary endpoints

The secondary objectives will be to evaluate the impact of a strategy of RASi continuation or discontinuation on per-anesthesia severe hypotension episodes, on postoperative mortality, and on episodes of acute kidney injury and hyperkalemia. Secondary endpoints will therefore be:

- episodes of hypotension requiring vasopressors administration during anesthesia and surgery. We define hypotension as a mean arterial pressure < 60 mmHg. All types of vasopressors will be considered (i.e. ephedrine, epinephrine, norepinephrine, or neosynephrine). Bolus and continuous infusion will be considered. Lowest arterial pressure, duration of hypotension, and total doses of vasopressors will also be collected and reported;
- episodes of hyperkalemia requiring therapeutic intervention;
- Acute kidney injury (according to the KDIGO criteria based on serum creatinine changes) [23];
- maximum SOFA (sequential organ failure assessment) score from postoperative day 1 to day 7 in patients admitted to ICU;
- duration of hospital stay (patients who will be outside the hospital but in other types of healthcare facilities at day 28 will be considered to have been discharged home);
- hospital-free days (censored at 28 days following surgery);
- all-cause mortality 28 days after randomization;
- Intensive Care Unit length of stay (when applicable); and
- hospital length of stay.

### Description of parameters for assessing efficacy endpoints

The primary endpoint is a composite endpoint, which will be collected at each visit. Diagnosis of complications will be made by investigators (anesthesiologists for medical complications and surgeons for surgical complications) and/or a specialist if he or she has been consulted for a complication. Because a double-blinded trial was not feasible (i.e. due to the higher number of medications containing an ACEi or ARB), an endpoint adjudication committee will review all events. Research assistants and experts of the adjudication committee will be blinded to the group assignment. Clinicians in charge of the patients will not be blinded. The endpoint adjudication committee will review clinical data and adjudicate efficacy endpoints while blinded to the assigned study drug group. The adjudication committee will be composed of physicians (cardiologists, neurologists, nephrologists, anesthesiologists) not participating into the study as investigators who will be provided with available collected data during the conduct of the study.

### Data management

All information required according to the protocol is entered in the case report forms. Data entry will be carried out on electronic media via a web browser. The data are collected as and when they are obtained and clearly recorded in these case report forms. Each missing data item must be coded. Clinical research associates (CRA) carry out regular follow-up visits at the study sites, after completing their initial visits. Monitoring the study will verify that the research patients are safe, protected, and their rights are being met, the data being recorded is accurate, complete, and consistent with the source documents, and the study is being carried out in accordance with the current version of the protocol, with all statutory and regulatory requirements. The investigator is responsible for the accuracy, quality, and relevance of all the data entered. In addition, the data are immediately verified as they are entered with consistency checks. The investigator must validate any changes to the values in the case report form. During or after the clinical study, the data collected about the research patients and sent to the sponsor by the investigators (or collaborators) will be anonymized. The anonymity of the patients will be ensured by reference to the first three letters of the name or initials of the full name of the person undergoing research on all documents related to the research protocol. Any suspected unexpected serious adverse reaction must also be declared electronically using the Eudravigilance European adverse drug reactions database managed by the European Medicines Agency (EMA). The sponsor must notify all the investigators any information that could adversely affect the safety of the participants. Assistance Publique Hôpitaux de Paris (AP-HP) is the sponsor of this study and has delegated power to its Clinical Research and Development Department (DRCD) in order to conduct the study in accordance with Article L.1121–1 of the French Public Health Code. AP-HP reserves the right to terminate the study at any time for medical or administrative reasons. In this case, the investigator will be informed accordingly.

**Fig. 3:**
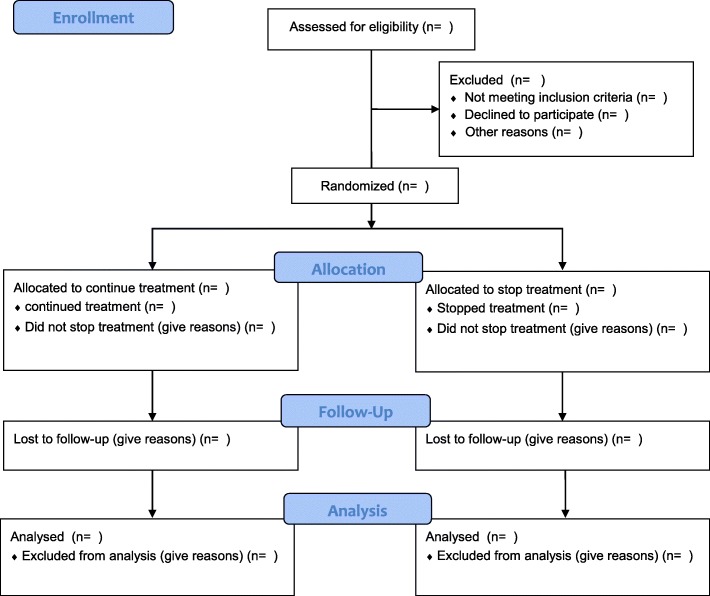
CONSORT *flow chart* of the study

In the case of lost-to-follow-up, the investigator will do his or her best to contact him or her in order to know whether he is alive. Mailing address, phone number, and phone number of at least one relative will be collected at inclusion to contact the patient. If an individual leaves the research prematurely, data relating to the participant can be used unless an objection was recorded when the patient signed the consent form. If consent is withdrawn, no data about the individual may be used unless he/she states in writing that he/she does not object. In practice, the participant is excluded from the research (Fig. 3 and Additional file 2).

It should be noted that all amendments will be validated by the institutional review board and, once validated, the content of the amendments will be communicated to all investigators through contact emails, newsletter (on a three-month basis), and during the investigators meeting.

### Description of statistical methods

Main analysis will be by intention-to-treat comparing the composite outcome measure at 28 days in the two groups by chi-squared test (or Fisher’s exact test as appropriate). Based on an incidence of 25% of the primary endpoint in the reference group, a total of 2222 patients will allow a 80% power to detect a 20% relative decrease of complications in the experimental group (corresponding to an absolute decrease of incidence of the primary endpoint of 5%) using a Chi-square test and considering that the two interim analyses will lead to a final test at a nominal alpha value of 0.0465 according to the O’Brien and Fleming principle. More detailed statistical analysis can be found in the Additional file 2. Sample size calculation was performed using the nQuery Sample Size Software (Statistical Solutions Ltd., Cork, Ireland), statistical analysis will be performed using SAS 9.4 (SAS Institute Inc., Cary, NC, USA).

## Discussion

In this RCT, we aim to evaluate the impact of a strategy of RASi continuation or discontinuation on perioperative complications in patients undergoing major non-cardiac surgery. Chronic treatment with RASi is highly prevalent in patients undergoing major surgery but lack of evidence has led to conflicting guidelines regarding the best management of these medications. The results of the trial should impact guidelines regarding management of RASi with higher level of evidence to anesthesiologists and surgeons before major non-cardiac surgery [24].

## Trial status

The first version was published on 15 December 2018 and was last updated on 2 July 2018 (including adjustment on the definition of major surgery). Study recruitment started on 6 February 2018. The estimated study completion date is 6 April 2021. AP-HP will be mentioned in the affiliation of the authors. Each site principal investigator will be listed as an author from the second author based on the number of patients included on each site (if at least one patient is included from the site). All other investigators will be listed as collaborators.

## Additional files

Additional file 1: SPIRIT 2013 Checklist: Recommended items to address in a clinical trial protocol and related documents*. (DOC 122 kb)

Additional file 2: Overview of the trial scheme for participants. (DOCX 28 kb)

## Abbreviations

ACE-Is: Angiotensin-converting enzyme inhibitors
AKI: Acute kidney injury
ARBs: Angiotensin receptor blockers
KDIGO: Kidney Disease Improving Global Outcomes
NYHA: New York Heart Association
RASi: Renin-angiotensin system inhibitors
