# Supplementary material for: Impact of renin-angiotensin system inhibitors continuation versus discontinuation on outcome after major surgery: protocol of a multicenter randomized, controlled trial (STOP-or-NOT trial)
Source: Trials. 2019 Mar 5;20:160. doi: 10.1186/s13063-019-3247-1 (PMC6402139; doi:10.1186/s13063-019-3247-1)
Supplement: Supplementary file 2 — Overview of the trial scheme for participants. (DOCX 28 kb) [file 13063_2019_3247_MOESM2_ESM.docx]

### Additional file 2.

**Definition of composite primary endpoint**: The primary endpoint will be met if one of the following complications occurs within 28 days from surgery (except for occurrence of Acute kidney injury which will be evaluated during the first 7 days).

- Death
- Acute Myocardial ischemia
- Arterial or venous thrombosis
- Stroke
- Acute pulmonary oedema
- Post-operative cardiogenic shock (requiring epinephrine or dobutamine infusion)
- Acute severe hypertension crisis (Defined as mean arterial pressure >130 mmHg)
- Cardiac arrhythmia (de novo atrial fibrillation, atrial flutter, ventricular tachycardia, ventricular fibrillation) requiring treatment
- Postoperative episode of sepsis (based on the sepsis 3 definition).
- Postoperative respiratory complication: defined by the need for intubation and/or noninvasive ventilation for respiratory failure,
- Need for unplanned intensive care unit admission or readmission
- Acute kidney injury: KDIGO criteria and renal replacement therapy. Baseline serum creatinine is obtained from the pre-operative blood sample.
- Surgical complications: need for reoperation for any reason and radiologic interventions for abscess drainage.
- Severe Hyperkalemia: serum potassium level >5.5 mmol/L and requiring therapeutic intervention (insulin/glucose infusion and/or sodium bicarbonate infusion and/or intravenous B2 agonists and/or intravenous calcium gluconate and/or renal replacement therapy and/or ventricular tachycardia or ventricular fibrillation).

### Definition of Secondary endpoints

- Episodes of hypotension (mean arterial pressure < 60 mmHg and/or requiring vasopressors) administration during anesthesia or surgery
- Maximum SOFA score from postoperative day 1 to day 7 in patients admitted to ICU
- Duration of hospital stay (patients who will be outside the hospital but in other types of health care facilities at day 28 will be considered to have been discharged home)
- Hospital free-days (censored at 28 days following surgery)
- All-cause mortality 28 days after randomization
- Intensive care unit length of stay (when applicable)
- Hospital length of stay

### Additional file 1.

**Statistical analysis**

We also plan to conduct multiple logistic regression analysis adjusting for baseline variables and stratification variables (heart failure history and center). Per-protocol analyses will be also conducted, especially when considering safety criteria (episodes of hypotension requiring vasopressors administration during anesthesia and surgery).

Primary analysis will be performed on all patients included and randomized in the trial in order to respect the intention-to-treat principle. Only patients who withdrew consent will be excluded.

Two interim analyses of main outcome measure will be performed after enrolment of one-third and two-third of the patients.

| No. of analysis | No. of subjects | Z | Type I error | P value |
| --- | --- | --- | --- | --- |
| Interim 1 | 741 | 2.79 | 0.0026 | 0.0026 |
| Interim 2 | 1481 | 2.29 | 0.011 | 0.0099 |
| Final | 2222 | 1.68 | 0.0465 | 0.0375 |

Subgroups analyses will be performed, in particular analyses will conducted according to patients’ comorbidities (history of heart failure or not, chronic renal failure), concomitant medication (beta-blocker, diuretics), pre-operative levels of biomarkers (natriuretic peptides), and subtypes for surgery.

Regarding secondary analyses, continuous variables will be compared using the unpaired t-test or the Mann Whitney U test and categorical variables will be compared using the chi-square test or the Fisher’s exact test.

Multiple regressions will also be conducted, seeking to identify independent risk factors of the composite outcome. The log-linearity of the quantitative variables will be systematically evaluated, and, if appropriate, variable transformations will be performed. A selection model process will be performed using stepwise selection method. The selection process model will be further validated by bootstrap. The existence of any colinearities will be observed, and a test of goodness of fit will be performed using the test and the van Cessie Houwelingen method. Finally a cross-validation procedure will be performed. The measures of association will be provided, with odds ratios and confidence intervals at 95 %.

To maintain an overall type I error at 5%, the critical level of significance of the interim analysis was fixed at according to O’Brien and Fleming principle. The trial will be stopped after interim analysis in case of significant difference between the groups or at the end of the planned patients’ enrolment.

Any missing value will be replaced by the previous value (the last value carried forward method). Sensitivity analyses for missing values will be conducted, for example, using a multiple imputation (MICE, multiple imputation by chained equations.
